# Supplementary material for: Are carbon emissions trading and green financial instruments synergistic? -Comprehensive quantitative research based on content analysis
Source: PLoS One. 2024 Mar 7;19(3):e0298601. doi: 10.1371/journal.pone.0298601 (PMC10919601; doi:10.1371/journal.pone.0298601)
Supplement: S1 Data — (ZIP) [file pone.0298601.s001.zip › Data/地级市Z_F工作报告.zip!/░▓╗╒/┬φ░░╔╜╩╨/└┤╫╘╣1⁄2╓┌║┼í╛┬φ┐╦╩2╛▌═°í┐_2017.docx]

# **政府工作报告（2017）**

【字体大小：[大](http://zwgk.mas.gov.cn/openness/detail/content/javascript:doZoom(18)) [中](http://zwgk.mas.gov.cn/openness/detail/content/javascript:doZoom(15)) [小](http://zwgk.mas.gov.cn/openness/detail/content/javascript:doZoom(14))】

各位代表：

现在，我代表市人民政府，向大会报告政府工作，请予审议，并请市政协委员和其他列席人员提出意见。

一、2016年工作回顾

过去的一年是我市非凡的一年，大事多、喜事多，同时难事也较多。我们面临经济下行、资源型城市转型以及洪涝灾害等多重挑战，在省委、省政府和市委的正确领导下，全市人民以新发展理念为引领，凝心聚力、克坚攻难，较好地完成了市十五届人大五次会议确定的主要目标任务，实现了“十三五”发展良好开局，向建市60周年献上了厚礼。

——经济指标实现新增长。整体经济走势呈现缓中趋稳、稳中有进态势。预计全市实现地区生产总值1440亿元，增长9%；财政收入222.75亿元，增长6.07%；固定资产投资2070亿元，增长11.3%；社会消费品零售总额470亿元，增长12.3%。地区生产总值增速超过全省平均水平。

——转型升级取得新成效。战略性新兴产业产值增长23%，占规上工业产值18%，同比提高2个百分点。国家级智能装备及机器人产业集聚区、省级轨道交通装备基地建设稳步推进，高端数控机床基地获批为省第二批战新试验基地。服务业占GDP比重达39.3%，提高1.8个百分点。金融、物流、电子商务、信息技术、工业设计、文化旅游等现代服务业加快集聚。

——创新能力实现新提升。创新实力跃居全省第4位，较上年上升1位；创新潜力跃居全省第2，较上年上升6位。综合能力跻身全省三甲，全社会研发经费占GDP比重2.63%，功效排名连续两年居全省第1。新登记注册企业6637户，增长19.4%。新增规上工业企业189家，总数达1184家。“四新”经济蓬勃发展。

——对外开放取得新突破。马鞍山综合保税区获得国务院批复。马鞍山口岸郑蒲港区正式对国际船舶开放，成为全省唯一的一江两岸同时对外开放的国家一类水运口岸。进口肉类指定口岸通过国家验收。马鞍山港完成货物吞吐量1.05亿吨，成为长江南京港以上第5个亿吨大港。对台合作势头良好，与台湾知名工商协会建立了全面战略合作关系，新增台资企业11家。

——民生福祉得到新改善。抗洪救灾全面胜利，灾后恢复和建设全面推进，420户受灾群众水毁房屋重建、修复任务提前完成。9680名贫困人口脱贫、2个贫困村出列的年度脱贫攻坚任务全面完成。33项民生工程和6件民生实事顺利办结。马鞍山长江大桥本地7座以下小型客车通行费减免元月一日开始实施。

回顾过去一年，成绩来之不易。这些成绩是在经济下行压力较大的背景下取得的,上半年我市GDP总量曾一度回落至全省第5位，增幅首次低于全省平均水平。这些成绩是在资源型城市转型升级压力较大的背景下取得的，由于去产能、实现脱困发展，马钢集团、华菱星马等重点企业生产增速较低。这些成绩是在我们遭遇60年一遇特大洪涝灾害的背景下取得的，洪涝灾害造成直接损失21.44亿元，间接损失更大。在困难和压力面前，各地区、各部门付出了极大辛苦。不仅这些成绩要倍加珍惜，进一步扩大和巩固，而且这种不畏艰难、知难而进的拼搏精神值得在新的一年工作中发扬光大。

一年来，我们主要做了以下工作：

（一）千方百计稳定经济增长。

为应对持续加大的下行压力，我们深入开展项目建设提速提效活动。认真落实“四督四保”要求，协同推进项目大谋划、大推进、大招商、大融资。全年完成省“大新专”亿元以上项目投资740亿元，占年计划的138.7%；华昌生物医药、乐通通讯等251个重点项目开工，华谊轻工名品、世界村橡塑新材料等260个重点项目加快建设，马钢埃斯科特钢、泰恩康药业等153个重点项目竣工投产，充分发挥了投资对稳增长的关键作用。深入推进服务企业活动。对重点企业实施“一企一组一策”帮扶，全年兑现产业扶持政策资金8.76亿元，落实结构性减税26.34亿元，减征缓缴社保费用4.8亿元，兑现稳岗补贴6919万元，惠及企业1100余家，支持了一批优势企业多产出、困难企业早脱困。加大招商引资力度。易佰特新能源、中兴科技产业园等30个10亿元以上大项目签约；实际利用省外亿元以上项目到位资金740亿元，增长11.1%；外商直接投资21.1亿美元，增长8.9%，招商引资总量及增幅均在全省保持领先。支持房地产市场健康发展。组合运用住房金融、公积金贷款、契税减免、购房补贴、棚改货币化补助等多种手段，促进房地产持续升温，全市商品房成交3.7万套、增长59.5%，金额182.8亿元、增长57.4%，有力地支撑了经济企稳。

（二）持续深入调整经济结构。

我们瞄准产业转型升级，强力推进国家和省级战新基地建设，明确市领导牵头，确定主导产业载体，配备工作专班。全市战新基地实现产值1172亿元，比规上工业产值增幅高12.5个百分点。支持传统产业改造升级，完成规上工业增加值610亿元，增长9%；规上工业企业实现利润83亿元、增长2.7倍，增幅居全省第一；马钢、华菱星马、蒙牛乳业、山鹰造纸等一批重点企业效益显著回升。加快现代服务业发展，入选2016年度“宽带中国”示范城市。马鞍山软件园、青年电商园发展势头良好。采石5A级旅游景区创建有序推进，大青山李白文化旅游区、鸡笼山景区晋升4A级。农业生产保持稳定，实现增加值90亿元、增长3.2%；新增市级农业产业化龙头企业61家、农民专业合作社199家、家庭农场316家，农业新型经营主体总量进一步壮大。县域经济发展活力增强，当涂县在全国百强县居74位，比上年提升2位。含山县、和县主要经济指标均保持两位数以上增长。

（三）系统实施创新驱动战略。

按照国家全创改的部署，我们着力培育创新主体，为290家企业发放“科技创新券”5868万元，新培育科技“小巨人”企业50家，新认定高新技术企业53家、总数超300家；设立产业升级基金20支，培育上市（挂牌）企业20家。加强创新平台建设，与南大、北大、上海交大等高校院所联合设立研发平台，新增省级企业技术中心10家，院士、博士后工作站各3家，国家机床产品质量监督检验中心通过验收；泰尔重工等3家企业通过国家技术创新示范企业认定，慈湖高新区获批全省首批知识产权示范园区。推动大众创业万众创新，马鞍山国家农业科技园入选全国首批“星创天地”，市科创中心获批国家小微企业双创示范基地，蓝海众创空间晋升国家级，富马智联创业园获批省级“青年创业园”，一大批创客走上创新创业之路。加大招才引智力度，引进高层次人才团队30家。国家质量强市示范城市通过预验收，新申报中国驰名商标4件，新增省著名商标38件。开发园区转型升级加速推进，市经开区、慈湖高新区、示范园区规上工业增加值均增长15%以上；当涂经开区创建国家级开发区报国务院待批，含山工业园扩区获省政府批复，宿马产业园建设稳步推进。

（四）坚定不移推进改革开放。

坚决去产能，配合马钢公司压减钢铁过剩产能139万吨，妥善安置员工1.33万人，“三供一业”移交工作有序推进。加快“放管服”改革，动态调整权责清单和涉企收费清单，编制市级公共服务清单和行政权力中介服务清单。深化商事制度改革，“五证合一、一照一码”全面实施。推进财政预决算公开，强化绩效管理，全面推开营改增试点。农村综合改革扎实推进，完成31万农户土地确权登记颁证和3个省级农村集体资产股份制改革试点任务。国有林场、供销社改革工作进展顺利。深化医药卫生体制综合改革，城市公立医院管理体制进一步理顺，基层医疗卫生体系建设进一步加强。行政机关公务用车、不动产统一登记、市县两级公共资源交易平台整合等改革全面完成。外经外贸工作成绩显著，完成进出口总额30.8亿美元，外经营业额3.5亿美元。外事侨务和对台工作取得新进展。成功举办海峡两岸电子信息博览会、物流论坛暨港航合作发展等重大展会，海峡两岸节能环保产业合作园落户我市。加入中德工业城市联盟，推进对欧经贸合作，全市欧洲企业达39家。坚持向东看，打好南京牌，努力全面融入长三角一体化发展。

（五）坚持不懈提升城市品质。

认真学习借鉴一、二线城市治理经验，打造更加宜居宜业新家园。完成城市总体规划修改，实现中心城区控制性详细规划全覆盖，城市设计水平进一步提升。对外交通加快建设，226省道二期工程基本完工，商合杭高铁含山段、347国道和县段、367省道、长江马鞍山段航道整治加快建设，郑蒲港铁路建设稳步推进。轨道交通1号线、合巢马城际铁路、九华路过江隧道等重大项目前期工作启动。水利工程建设稳步推进，防洪薄弱环节治理项目全面启动，水阳江下游、裕溪河等重要支流和中小河流防洪治理工程有序推进。加强城镇基础设施建设，慈湖河路改造一期建成通车，江东大道南延工程加快建设，红旗路贯通工程开工。美丽乡村建设取得新成效，全面推进16个乡镇政府驻地建成区和36个中心村整治工作，黄池镇入选全省首批特色小镇。成功创建省级生态县区2个、乡镇4个。PM10平均浓度75微克/立方米、下降13.8%，降幅居全省前列。节能减排任务全面完成，单位生产总值能耗下降4%，主要污染物排放总量控制在省下达指标内。我们的城市天更蓝、水更清、山更绿。

（六）尽心尽力保障改善民生。

在财力紧张的情况下，保障民生力度持续加大，全年民生支出178.25亿元，增长5.5%，占财政支出的83.2%。大力促进就业创业，城镇新增就业5.66万人，期末城镇登记失业率为2.94%。全面实施机关事业单位养老保险制度，“全民参保登记计划”全国试点工作持续推进，新型农村合作医疗、大病保险制度实现全覆盖。城乡低保人均月保障标准分别增长5%和8%。城市社区助老服务站全部建立，农村社区养老服务站全部联网，新增社会办养老机构12家。推进卫生计生服务均等化，实施12项国家基本公共卫生服务，新增4项市级基本公共卫生服务，成为全国首批、全省唯一的“健康城市试点市”。建成各类保障性安居工程1.3万套，实施老旧小区整治工程25个。加大义务教育投入，新改扩建公办幼儿园13所，完成25所义务教育学校标准化建设，教学点在线课堂实现全覆盖。通过国家公共文化服务标准化试点评估验收，在全国率先建成数字文化体验馆，继续办好李白诗歌节等群众性文化活动。广泛开展全民健身运动，举办大型体育赛事23场。我市运动员孟苏平在里约奥运会上勇夺金牌，成为我市第二位、安徽第三位奥运冠军。新闻出版、广播电视、文学艺术、社会科学、档案和地方志工作水平不断提升。工会、共青团、妇女儿童、残疾人、红十字事业取得新进步，气象、地震、防灾减灾工作取得新进展。

（七）持续创新社会治理工程。

加强城乡社区建设，创建“精品”、“四优”社区60个，社会服务管理信息化平台上线运行。深化社区矫正、安置帮教、人民调解工作，城乡公共法律服务体系实现全覆盖。扎实推进村居工作者管理平台和村居“大管家”服务平台建设，打通服务群众“最后一公里”。开展信访积案化解行动，信访法治化深入推进，信访总量下降12.3%，进京非访下降90%。社会治安防控体系整体水平有效提升，刑事案件发案数下降18%，公安部门多项工作走在全省前列。深入开展安全隐患排查“百日攻坚”活动，安全生产教育培训和隐患排查工作全省领先，没有发生较大以上生产安全事故，事故总量得到有效遏制。强化食品药品安全监管体系和能力建设，启动国家食品安全示范城市创建工作。第三次全国农业普查取得阶段性成果。粮食安全责任制全面落实。民族团结、宗教和睦局面进一步巩固。推动军民深度融合发展，实现“全国双拥模范城市”称号“三连冠”，人民防空军事斗争准备检验评估获全省第一，国防教育、国防动员工作得到加强，驻马部队和民兵预备役人员在支持经济发展、促进社会稳定特别是抗洪抢险救灾等方面作出了突出贡献。

过去的一年，我们不断强化政府自身建设。坚持把树牢“四个意识”作为根本遵循，始终与以习近平同志为核心的党中央保持高度一致；坚持把致力发展作为根本任务，一心一意谋发展，集中精力搞建设；坚持把履职尽责作为根本要求，开拓进取，知难而进，敢于担当，勇于负责；坚持把依法行政作为根本保障，主动向市人大及其常委会报告工作，向市政协通报情况，办理市人大议案4件、建议111件，市政协提案280件；坚持把纪律和规矩作为根本准绳，推进审计监督全覆盖，严肃执纪问责。

各位代表！

过去一年取得的成绩，是省委、省政府和市委坚强领导的结果，是市人大、市政协大力支持的结果，是历届班子扎实苦干的结果，是全市上下团结奋斗、锐意进取的结果。在此，我代表市人民政府，向全市人民，向广大干部群众，向市人大代表、市政协委员、离退休老同志，向市各民主党派、工商联、无党派人士、人民团体和各界人士，向中央和省驻马单位、人民解放军、武警官兵和人民警察，向所有关心、支持、参与马鞍山建设发展的境内外投资者、海内外朋友，表示衷心的感谢和崇高的敬意！

回顾总结去年的工作，需要特别报告的是，去年全市城市居民人均可支配收入增长7%，未能实现8%的预期目标；农村居民人均可支配收入增长8.5%，未能实现9%的预期目标。究其原因，主观上是我们的工作努力不够；客观上是受部分国企脱困发展、减员增效以及洪涝灾害影响所致。同时，我们也清醒地认识到，前进道路上还存在不少困难和问题：民营企业投资意愿不强，经济企稳的基础不牢；战略性新兴产业集聚度不高，大项目、好项目偏少；财政收支矛盾突出，政府负债率偏高；公共服务体系不尽完善，民生工作与群众期盼还有差距；政府工作还存在不足，有些地区和部门工作拖沓，敷衍塞责；少数干部不作为、不会为，甚至乱作为。对此，我们一定增强忧患意识和担当精神，采取切实措施加以防范和化解，决不辜负全市人民的期望和重托。

二、2017年工作总体部署

今年是党的十九大召开之年，是实施“十三五”规划的重要一年，做好今年政府各项工作意义重大。展望全年，我们站在了一个新的历史起点，面临许多新的历史重大任务，需要我们不忘初心，逐梦前行。既要坚定不移保持经济发展中高速，努力走在全省的前列，缩小与发达地区的差距；又要坚定不移地推进供给侧结构性改革，适应新的消费需求，提高供给的质量和效益，实现产业层次中高端。既要坚定不移实施创新驱动，全面深化改革创新，培育自主创新能力，培植经济发展新动能；又要坚定不移实施开放联动发展，推进重大基础设施建设，加速融入南京都市圈、合肥都市圈，拓展经济发展新空间。

综合各方面情况，做好今年工作，我们依然面临许多困难和挑战。国际上不确定、不稳定性因素将进一步凸显；国内结构性供需矛盾失衡、金融和实体经济失衡、房地产和实体经济失衡等等，都可能对我们的发展造成影响和冲击。但辩证地分析，今年我们也面临许多难得的发展机遇和有利条件。从全国看，中央宏观调控政策更加务实精准，比如研究新设一些自贸区、加快智能制造示范区建设、加强财产权保护、完善支持资源枯竭型城市转型、允许地方政府在法定权限范围内制定招商引资优惠政策等，都为我们发展提供了新的更大支持。从长三角看，长三角城市群规划已正式实施，我市作为安徽的东大门、对接的桥头堡，是等高对接苏浙沪的最先一公里，有基础、有条件抢占新一轮发展先机，加速与南京等地一体化发展。从全省看，省委、省政府对我市提出了“三个走在前列”、“做好五篇文章”的殷切期望，将芜马城市组群定位为国家民族企业创新平台、长三角乃至全国的钢铁工业基地和先进制造业基地、生态宜居型滨江都会区，积极推进合芜马共同申报国家级自贸区，我市在皖江地区乃至全省的战略地位进一步提升。从自身看，工业基础雄厚，产业配套齐全，科技创新能力强，生态环境优美，在承接国内外产业转移方面综合优势明显。总之，困难和挑战并不可怕，没有过不去的坎，只要我们坚定必胜信心，迎难而上，就一定能够战胜困难，完成全年经济社会发展任务。

今年政府工作的总体要求是：全面贯彻党的十八大和十八届三中、四中、五中、六中全会精神，深入贯彻习近平总书记系列重要讲话特别是视察安徽重要讲话精神，全力推动“三个走在前列”，坚持稳中求进、进中求好、好中求快，以提高经济发展质量和效益为中心，以推进供给侧结构性改革为主线，以转型升级、加快发展为主题，以五大发展行动计划为总抓手，大力实施创新驱动发展战略、开放带动战略，全面做好稳增长、促改革、调结构、惠民生、防风险各项工作，奋力打造经济强市、文化强市、生态强市，保持经济平稳健康较快发展和社会和谐稳定，以优异成绩迎接党的十九大胜利召开。

综合考虑，今年全市经济社会发展的主要预期目标为：地区生产总值增长9%，财政收入增长6%，固定资产投资增长11%，社会消费品零售总额增长11%，城镇居民人均可支配收入增长7.5%，农村居民人均可支配收入增长9%，城镇登记失业率控制在4%以内，单位GDP能耗、主要污染物排放总量控制在省下达指标以内。

做好今年政府工作，要做到“四个更加注重”：

一是更加注重“三个走在前列”的总目标。“三个走在前列”是省委赋予我们的重大历史使命，与我市“全省争三强、人均居前列、建设更高水平小康社会”奋斗目标高度契合。我们要强化责任担当，牢记使命、不辱使命。各项工作都要按照“走在前列”的要求，自我加压、提升标杆，一方面对已居全省前列的指标继续保持领先；另一方面对于没达到全省平均水平的指标要力争比学赶超。

二是更加注重“五大发展行动”的总引领。岁末年初，市委、市政府制定了五大发展行动计划，以及制造业升级、文化强市、生态强市行动计划，这是我市转型升级、加快发展的一揽子行动纲领。我们要围绕行动计划确定的36项重大工程，一个一个排出施工图、时间表、责任书，确保今年一批重大工程扎扎实实开局。

三是更加注重供给侧结构性改革的总统筹。推进供给侧结构性改革对我市转型发展意义尤为重大。目前钢铁产业过剩局面没有根本改变，仍处于深度调整中，去产能的决心不能动摇，力度不能减弱。我们要增强定力、巩固成果、乘势而上，加力抓好今年“三去一降一补”工作。

四是更加注重稳中求进的总基调。首先要稳，稳是基础。稳态势、稳政策、稳大局，坚决守住金融风险、社会民生、安全生产、生态环保的红线、底线；同时要坚持积极进取，在稳的前提下，力争各项工作一步一个脚印向前迈进，取得新进展、实现新突破。

三、2017年重点工作安排

今年经济社会发展任务十分繁重，各项工作都要抓早抓紧、抓实抓好，既要统筹兼顾，又要突出重点。着重做好八个方面的工作：

（一）狠抓项目建设，保持经济平稳较快发展。

面对复杂多变的宏观环境，稳增长仍然是各项工作的重中之重，把项目建设作为稳增长、调结构、增后劲的总抓手，努力争取经济增长达到或超过预期目标。

开展“项目建设年”活动。围绕全市重大战略、重点领域和优势产业，抓好项目谋划。力争全市重大项目库规模达2000亿元，其中支撑要件完善的项目规模达1000亿元，争取更多项目纳入国家和省里盘子。坚持条块结合、分级调度，抓好项目推进。开工建设安积电半导体制造、泰盛高档生活用纸等230个重点项目，建成投产裕祥高端数控机床、瑞声科技等170个重点项目，完成投资870亿元。其中市级重点调度的120个重点项目完成投资350亿元。切实抓好项目征地、拆迁、供地和资金保障工作。

全力以赴抓好招商引资。坚持领导干部带头招商、一线招商，强化招商组织体系、完善招商政策体系、改进招商工作体系、优化招商考核体系，重点面向央企、知名民企、世界500强和港澳台企业进行招商，着力招大引强。充分发挥各载体招商机构、战新产业招商小组，以及市派招商办事处主力军作用。确保今年全市续建和新建招商引资工业项目完成投资400亿元，实际利用省外亿元以上项目到位资金810亿元、外商直接投资22.9亿美元。

大力支持实体经济发展。继续开展精准帮扶企业活动，一企一组一策，建立问题清单、责任清单、服务清单，切实帮助企业解决实际问题。支持马钢做精钢铁主业、发展多元产业、培育战新产业、引领创新创业，开创转型升级、健康发展新局面。支持华菱星马深化改革、开拓市场、扩大合作，带动全市汽车产业做大做强。支持山鹰纸业加快150万吨造纸项目建设。持续做好为中小微企业服务工作，抓好降成本20条政策落实，进一步在减税降费和降低人工、用地、用能等要素成本上加力增效。完善"4321"新型政银担风险分担合作机制，继续实施中小微企业还贷周转金等政策。

保持房地产市场健康发展。修改完善土地开发管理办法、购房契税减免等政策；严控商业用地供应，引导商业用房进行结构调整，改造为科技孵化器、众创空间等，进一步加大房地产去库存力度。继续推进棚改货币化安置，完善农民工、外来务工人员市民化配套政策，规范发展住房租赁市场，促进房地产市场平稳健康发展。

（二）实施创新驱动，推动经济转型升级。

产业转型升级是我市一项重大而紧迫的任务。必须坚持把创新作为第一动力，积极抢抓“全创改”机遇，构建创新型现代产业体系。

促进产业结构优化升级。坚持以“三重一创”为引领，加快推进国家级智能装备（机器人）和省级轨道交通装备、高端数控机床3个战新基地建设，力争全市战新产业产值增长15%。加快传统产业升级改造，把智能制造作为两化融合的主攻方向，实施亿元以上技改项目100个、完成投资400亿元。大力发展金融保险、现代物流、检验检测、工业设计、科技服务等生产性服务业，文化旅游、健康养老等新兴服务业；改造提升传统服务业，加快传统商圈提档升级，打造特色街区，培育新的消费增长点，满足个性化、多样化消费需求。力争服务业占GDP比重超过40%。

增强企业自主创新能力。依托马钢、安工大、中钢矿院等本地创新主力军团，通过开展国际合作、深化军民融合、引进大院大所等举措，加快科技成果在我市实现产业化。加强科技创新平台建设。大力支持政产学研用合作，组建工程技术研究中心、重点实验室等研发平台。充分发挥科技创新券、科技奖励政策等引导作用，加大对有技术支撑、有市场前景的科技型企业培育力度，新培育科技“小巨人”企业50家、高新技术企业50家，实现高新技术产业增加值187亿元。

强化创新体制机制建设。深入实施人才集聚"313"行动计划，力争全年培育引进高层次科技人才创业团队30个、企业创新团队20个，培养优秀企业家20名、“两高人才”6000名。实施青年创新创业计划，发展众创、众包、众扶、众筹等新模式，构建“众创空间+孵化器+加速器+产业基地”一体化孵化体系，掀起大众创业万众创新新热潮。深化科技金融融合，积极培育引进各类产业投资基金，推进产业链、创新链、资金链精准对接。实施首担、首贷、首投、首补，帮助创新创业人员解决“第一桶金”。

（三）持续深化改革，增强经济发展动能活力。

深化改革是我市转型升级、加快发展的根本途径。必须加快破除体制机制障碍，激发经济发展内生动力和活力。

深化“放管服”改革。动态完善权责清单、涉企收费清单，全面实施公共服务清单和中介服务清单。完善事中事后监管措施，全面推开“双随机、一公开”模式。深化商事制度改革，扩大“证照合一”覆盖面。深化人才体制机制改革，建立高层次人才柔性引进机制，加大急需紧缺人才引进力度；鼓励高校院所科研人员创新创业和自由流动。积极探索完善党政机关与企事业单位人才、市直机关与基层公务员流动机制。

深化国企国资改革。推进国有企业完善公司法人治理结构，建立企业领导人员分层管理制度，完善激励约束机制。规范国有融资平台融资行为，建立风险预警机制，防范债务风险。鼓励发展民营资本控股或参股国有企业，鼓励国有企业之间相互合作。全力支持马钢去产能，配合做好职工安置，推进企业办社会职能分离移交工作。强化国有投资和股权监管，提升国有资本配置和运行效率。

深化财税金融改革。运用积极的财政政策，着力厚植财源，涵养税源；加强收入征管，提高收入质量，努力完成预算收入任务；加强支出管理，最大限度发挥财政资金使用效益。深化国税、地税征管体制改革。在继续扩大信贷规模的同时，采取资产证券化、股权投资、债权投资、投资基金、PPP合作等方式扩大直接融资规模。完成企业上市挂牌5家、辅导在审5家。大力发展消费金融、汽车金融、融资租赁等新型金融业态。

大力发展民营经济。坚持“两个毫不动摇”，全面落实支持民营经济发展各项政策。试行企业投资项目管理负面清单制度，探索“不再审批”管理模式，对符合条件的企业试行投资项目备案承诺制。加大PPP项目推进力度，在产业培育、基础设施、医疗卫生、生态建设等方面谋划推出一批示范合作项目，争取成为国家PPP项目实施试点市。加强企业家队伍建设，稳定民营企业家信心，构建“亲”“清”新型政商关系。

（四）开放联动发展，打造对外开放高地。

 马鞍山处在皖江开放最前沿，必须围绕开放联动走在全省前列要求，加大对内对外开放力度，全面提升开放型经济发展水平。

 深入推进以港兴市。全力支持郑蒲港新区跨越式发展，建设“港产城”一体的现代化新城区；将郑蒲港打造为长江经济带的重要节点港、安徽江海联运枢纽港以及沿海国际大港的喂给港。加快综保区建设，确保今年顺利通过验收，实现封关运行。开埠运营肉类指定口岸，加快粮食、整车、冰鲜等指定口岸申建，积极参与安徽自贸区申报。整合全市港口岸线资源，做好岸线资源保护、建设和管理工作。深化与盐田港、舟山港、合肥港合作，积极与省内城市共建无水港。大力引进知名船代、货代、航运企业，吸引更多货物从我市集并中转，力争完成港口吞吐量1.1亿吨、集装箱25万标箱。

 全面扩大对外开放。推进大通关建设，深化关检“三个一”合作，提高港口现代化管理水平。鼓励马钢、十七冶、建诚国际等企业走出去，推动外贸进出口总量和外经营业额继续走在全省前列。强化对台合作，密切马台经贸往来，加速台资企业集聚，打造台商投资高地。强化对欧合作，积极创建中欧合作产业园。推进与苏浙沪等高对接，加强科技、信息、金融、人才、市场等发展要素共用共享。推动芜马同城化，支持宿马产业园建设。
推进园区转型升级。园区是经济建设主战场、经济发展的主引擎，必须面向国际国内两个市场、两个资源，推动园区转型升级发展。突出首位产业、主导产业、未来产业，错位发展、差异化竞争，提高产业集聚度。加快推进开发区目录修订完善，力争我市10个开发区在国家和省级目录内。运用“互联网+”，加大研发投入，培育更多高新技术企业。实施质量品牌升级工程，提高产品质量。加大对外合作力度，面向国内外市场，实施兼并重组联合，盘活资产存量、吸引投资增量。

 （五）统筹协调发展，推进新型城镇化建设。

 补齐短板，提升内涵，是提升我市城市发展水平的关键所在。必须统筹抓好城乡规划、建设和管理工作。

 打造通畅便捷交通体系。推动轨道交通1号线、宁和城际项目前期取得实质性进展。加快合巢马城际铁路项目前期，争取早日报批。协调苏340省道、苏341省道加快建设，力争尽快与我市马濮旅游大道、314省道对接工程竣工通车。推进247省道前期工作，加快367省道建设。推动一江两岸协调发展，加快九华路过江隧道前期工作，推进商合杭高铁含山段建设，启动329国道项目前期，加快347国道和县段建设。改造400公里农村道路，推进农村客运公交化改造。

 提升城镇功能内涵品质。突出规划引领作用，启动2030年城市总体规划修编，推进城市设计省级试点，开展控规动态维护，完善专项规划，探索“多规合一”。加强城区路网建设，续建慈湖河路改造、江东大道南延、九华东路二期工程，实施银杏大道东延项目。加强城市污水处理厂配套管网建设，开展城市易涝点整治。深入推进城市管理执法体制改革，整合市政公用、市容环卫、园林绿化、城市管理执法等相关职能，不断提高城市管理精细化、智能化水平。推进智慧城市建设，加强无线城市、宽带提速、三网融合等信息基础设施服务能力建设。大力推进美丽乡村建设，着力打造特色小镇。

 加快发展现代农业。强化县域功能，改善人居环境，提升县城品位，推进新型城镇化。大力发展现代都市农业，把增加绿色优质农产品供给放在更加突出位置，狠抓农产品标准化生产、品牌创建、质量安全监管。着力培育新型农业经营主体和服务主体，新增市级示范家庭农场30个、农民专业合作示范社20个。实施农村土地所有权、承包权、经营权分置改革，积极推进农村集体资产股份合作制改革和资源变资产、资产变股金、农民变股东改革试点。

 （六）推进绿色发展，加快建设生态强市。

 良好生态环境是马鞍山最宝贵的财富。必须深入推进绿色发展、低碳发展、循环发展，着力打造山清水秀、天蓝地绿的美丽家园。

 加强生态文明建设。实施生态强市建设行动计划，指导县区创建省级和国家级生态文明示范区，推进十大重点生态项目，继续夯实生态市创建基础。启动国家森林城市创建，坚持高水平规划、高标准建设，着力增加绿化总量，提升绿化品位档次。新增造林5万亩，森林覆盖率达28%以上。加快餐厨垃圾处理项目建设，确保垃圾焚烧发电项目建成投产。
持续改善环境质量。强化大气污染防治，加强重点行业脱硫脱硝除尘设施建设，在火电、造纸行业实行企业按量排污许可制度，强化城市扬尘综合治理，加强秸秆禁烧和综合利用，加快黄标车淘汰，完善大气质量监测预警体系，确保全年PM10平均浓度持续下降。开展饮用水水源地规范化建设，实现从水源到水龙头全过程监管。加大环境执法监管力度，坚决打击各类环境违法行为。

 节约高效利用资源。大力发展节能环保产业，推进茂迪太阳能电池及组件制造、银欣科技等新能源项目建设。严格实施能源消费总量和强度双控制度，开展煤电节能减排升级改造计划，清洁高效利用化石能源，大力发展可再生能源。积极推广建筑产业化和绿色建筑建材。开展全民节能行动计划，加强重点领域和公共机构节能，推行合同能源管理。深入推进国土“四模创建”，大力实施建设用地总量和强度“双控”行动，继续开展批而未供、供而未用土地清理处置工作。强化用水定额管理，提高水资源配置和利用效率。

 （七）打造文化强市，彰显文明城市魅力。

 文化是区域发展的重要竞争力，更是人民群众的精神家园。必须深入实施文化强市建设行动计划，巩固提升全国文明城市创建成果。

 大力发展文化事业。持续推进公共文化服务标准化建设，完善公共文化服务体系，新建10个乡镇、100个村（社区）综合性文化服务中心，打造全民阅读名城、数字文化名城、文化旅游名城。创新举办第29届李白诗歌节，打造李白文化品牌，叫响中国诗歌之城。积极承办2017年中国文化馆年会。推广“互联网+文化”服务模式，完善“文化有约”服务平台。加强文物非遗保护，推进凌家滩国家考古遗址公园等项目建设。繁荣发展哲学社会科学，进一步做好地方志工作。

 加快发展文旅产业。抓好景区创建，深化采石风景区管理体制改革，完成采石创建5A级旅游景区公共服务体系建设，推动褒禅山香泉度假区争创省级旅游度假区，新增2个4A级旅游景区。创新营销方式，开拓周边市场，力争旅游收入和人数增长超20%。支持含山争创国家全域旅游示范区。推进创客加文创园等园区建设，引导文化产业集聚发展。大力开展全民健身运动，举办知名展会和文体赛事，推动体育产业蓬勃发展。

 打造文明城市升级版。积极培育社会主义核心价值观，加强未成年人思想道德建设，提升公民科学素质，广泛开展志愿服务，推进社会诚信体系建设。开展文明创建“四整治四提升”行动，推进文明创建常态化、长效化。

 （八）加强民生建设，共建共享发展成果。

 让人民群众过上幸福美满的生活，是政府工作的出发点和落脚点。必须自觉践行以人民为中心的发展思想，保障基本民生，提升基本公共服务水平。

 打赢脱贫攻坚战。落实源头严把、过程严控、验收严格、纪律严明的“四严”要求，建立完善全覆盖的精准扶贫、监测评估、防范返贫、责任落实四项机制，确保完成剩下的10227人脱贫、26个贫困村出列的任务，在全省率先打赢脱贫攻坚战。完善城乡最低生活保障和社会救助制度，努力实现对困难群众的精准救助。重视关爱留守妇女儿童，维护残疾人合法权益，积极帮扶困难职工和农民工。

  增强社会保障能力。强化公共就业服务，城镇新增就业4.3万人。实施大学生就业创业促进计划，吸引更多大学生来我市就业创业。深入推进“全面参保登记计划”全国试点，扩大社会保险覆盖面。启动养老服务体系建设三年行动计划，加快建设社区养老服务设施，改造提升农村敬老院设施设备，支持社会力量兴办医养结合型养老机构，全面提升我市养老服务水平。

 加快社会事业发展。大力发展更高质量更加公平的教育，力争普惠制幼儿园覆盖面超85%，全面改善农村薄弱学校办学条件，稳妥推进普通高中课程改革和考试评价制度改革；加强现代职业教育体系建设，推进技工大市建设。深化城市公立医院改革，加快医联体和分级诊疗体系建设，进一步解决看病难、看病贵问题。推进健康马鞍山创建，命名一批健康社区、健康村镇、健康单位、健康家庭。强化公共卫生和重大疾病防控。改革完善计生服务管理。夯实统计基层基础，认真做好第三次全国农业普查。继续做好外事、侨务等工作。

 提升社会治理水平。深化和谐社区建设，加强城乡社区协商。加大社会组织培育力度，完成行业协会商会与行政机关脱钩。深化信访工作制度改革，全面推进“阳光信访、责任信访、法治信访”。完善社会治安防控体系，严密防范和依法打击各类违法犯罪行为。加强互联网安全管理，构建和谐文明网络空间。牢固树立“依法治安”理念，强化安全生产责任制落实，巩固隐患排查“百日攻坚”成果，有效防范、坚决遏制较大以上安全事故发生。深入实施食品药品风险分级监督管理，扎实做好国家食品安全示范城市创建工作。落实粮食安全责任制。全面完成气象现代化建设任务。维护民族团结和宗教和谐稳定。做好国防教育、国防动员、人民防空和双拥优抚安置工作。

 各位代表！今年的政府工作蓝图已绘就，本次代表大会审议通过后，将成为摆在各级政府面前的一份庄严考卷。我们深知天上不会掉馅饼，努力奋斗才会梦想成真。我们将时刻牢记习近平总书记的教导，加强政府自身建设，提高政府执行力，撸起袖子加油干，努力向全市人民交上一份圆满的答卷。要只争朝夕快干。以时不我待的紧迫感，抓紧每一天、干好每件事。对每项政府工作，制定时间表、路线图、施工图，挂图作战，同时压实责任，推进工作任务菜单式分解、责任契约式落实、绩效审计式验收、结果问责式考核。要知难而进苦干。今年的工作大事多、难事多、要求高，面临的困难可能更多，挑战可能更严峻，我们必须敢于啃硬骨头，敢于迎难而上，把该担的责任担起来，把该抓的工作抓到位，埋头苦干。要勤勉履职实干。恪尽职守，夙夜在公，主动作为，善谋勇为。抓住想干事、敢干事两个关键点，完善考核评价体系，健全正向激励和容错机制，坚决整肃懒政怠政行为，决不允许为官不为。要遵规守纪正干。带头尊法学法守法用法，严格依照法定权限和程序履行职责，习惯运用法治思维和方式推动工作。自觉接受市人大法律监督、工作监督和市政协民主监督，支持工会、共青团、妇联等群团组织开展工作。认真落实党风廉政建设主体责任，强化党内政治生活。严厉整治各种违规违纪行为，坚决纠正侵害群众利益的不正之风，坚定不移惩治腐败，着力营造风清气正的政治生态。

 各位代表！

 努力奋斗才能梦想成真。让我们紧密团结在以习近平同志为核心的党中央周围，在省委、省政府和市委的坚强领导下，开拓创新，务实苦干，圆满完成今年政府各项任务，为全力推动“三个走在前列”、实现马鞍山新一轮大发展大提升、建设更高水平小康社会作出新贡献，以优异的成绩迎接党的十九大胜利召开！
